# Integrated multi-omics analysis uncovers cervicovaginal ecological networks and their association with *Chlamydia trachomatis* load

**DOI:** 10.1128/iai.00681-25

**Published:** 2026-06-12

**Authors:** Caleb M. Ardizzone, John W. Lammons, Renny S. Lan, Jacob H. Elnaggar, Rebecca A. Lillis, Evelyn Toh, Lindsay M. Pack, Patricia D. Mott, Clayton D. Jacobs, Laxmi Yeruva, Christopher M. Taylor, Alison J. Quayle

**Affiliations:** 1 Department of Microbiology, Immunology, and Parasitology, Louisiana State University Health Sciences Center 12258, New Orleans, Louisiana, USA; 2 Department of Microbiology and Immunology, Indiana University School of Medicine 12250, Indianapolis, Indiana, USA; 3 Department of Pediatrics, University of Arkansas Medical Center 12215, Little Rock, Arkansas, USA; 4 Microbiome and Metabolism Research Unit, Arkansas Children’s Nutrition Center, Little Rock, Arkansas, USA; 5 Department of Medicine, Division of Infectious Diseases, Louisiana State University Health Sciences Center 12258, New Orleans, Louisiana, USA; University of Virginia, Charlottesville, Virginia, USA

**Keywords:** bacterial vaginosis, *Chlamydia trachomatis*, cytokines, tryptophan metabolome, vaginal microbiome, women

## Abstract

*Chlamydia trachomatis* (Ct) is a causal agent of upper reproductive tract pathology. There is a broad spectrum of cervical Ct load in infected women, and upper tract infection is associated with higher cervical Ct load. Recent studies indicate that bacterial vaginosis (BV) can modulate host-Ct outcomes. To identify features associated with BV status and Ct load, we performed an integrated multi-omics analysis of the cervicovaginal microbiome, tryptophan metabolome, and cytokines. Samples were analyzed using 16S rRNA gene sequencing, targeted UPLC-MS/MS quantification of tryptophan metabolites, and multiplex cytokine profiling. Ordination analyses showed that BV status was separated by the microbiome, metabolome, and cytokines, whereas Ct load was separated only by cytokines. K-means clustering of tryptophan metabolites defined three metabolome state types (MSTs). MST I, associated primarily with *Lactobacillus crispatus*-dominated community state type (CST) I, exhibited high tryptophan availability, indole-3-lactic acid, and complete kynurenine-pathway activity. Both MST II and MST III were associated with BV-associated CST IV and showed marked tryptophan depletion. MST II was broadly depleted of most tryptophan metabolites, while MST III was enriched in downstream microbially derived indole pathway metabolites and kynurenic acid. Hierarchical all-against-all association testing revealed coordinated relationships linking clusters of bacterial taxa, metabolites, and cytokines. Importantly, multi-omics network analyses identified integrated microbial-metabolic-immune modules that predicted high versus low Ct load, highlighting CXCL9, CXCL10, IL-17, BV-associated taxa, and indole pathway metabolites as key discriminative features. Results demonstrate that cervical Ct load reflects coordinated microbial-metabolic-immune ecological states rather than microbiome composition alone and refine current models of Ct-BV interactions.

## INTRODUCTION

*Chlamydia trachomatis* (Ct) infection in women is predominantly asymptomatic and chronic, taking months to resolve. The cervical epithelium is the primary reservoir of Ct, and infection can ascend and cause severe upper reproductive tract sequelae (1). There is an extremely broad spectrum of cervical Ct load in infected women (2), and upper tract infection is associated with higher cervical Ct load (3, 4). Defining the genital milieu that supports high burden reservoirs or, conversely, permits natural clearance of Ct could inform clinical care guidelines and how immunity operates or is impeded in this unique site.

As an obligate, intracellular bacterium, Ct is dependent upon the resources available in its epithelial niche. As such, Ct has evolved multiple virulence factors to counter effector immune mechanisms and to acquire essential nutrients from its host (5–7). One central factor in the host-Ct interplay is the cytokine interferon gamma (IFNγ), which induces the tryptophan-catabolizing enzyme indoleamine-2,3-dioxygenase 1 (IDO1) (8). At sufficient and sustained concentrations *in vitro*, IFNγ can eradicate Ct, a tryptophan auxotroph (9). At sub-optimal IFNγ concentration, however, Ct can enter into a viable but non-replicating persistent form until favorable tryptophan conditions enable it to restart replication (10–12). Germane to IFNγ-induced starvation, genital Ct serovars have a functional tryptophan synthase that enables them to synthesize tryptophan via indole salvage, potentially evading eradication (13, 14). Since neither Ct nor humans synthesize indole, the source of this tryptophan catabolite is hypothesized to be the bacteria associated with bacterial vaginosis (BV) (13, 15).

BV is characterized by a loss of lactobacilli and an overgrowth of opportunistic anaerobic bacteria and is highly prevalent in Ct-infected women (2, 16–19). We and others have reported that women with BV are less likely to naturally clear Ct infection, but results are quite varied, and this could be explained by the multiple ways of classifying BV and BV treatment outcomes and/or depletion or abundance of specific bacterial types across the spectrum of vaginal dysbiosis (2, 17, 20). Further, dysbiotic microbiomes are likely to modulate tryptophan metabolism and impact Ct survival and growth in ways we do not yet understand. For example, several microbial-produced tryptophan metabolites can induce Ct tryptophan synthase even during tryptophan sufficiency (21), and growing evidence indicates that many tryptophan metabolites have immunomodulatory functions and play a major role in homeostasis at other mucosal sites (reviewed in references 22–24).

Here, integrative multi-omics analysis reveals that vaginal microbial composition, tryptophan metabolism, and mucosal cytokines form interconnected ecological networks and that variation in Ct load aligns most clearly with coordinated microbial-metabolic-immune states rather than microbial composition alone or any single factor.

## RESULTS

### Cohort and specimens

**Fig 1:**
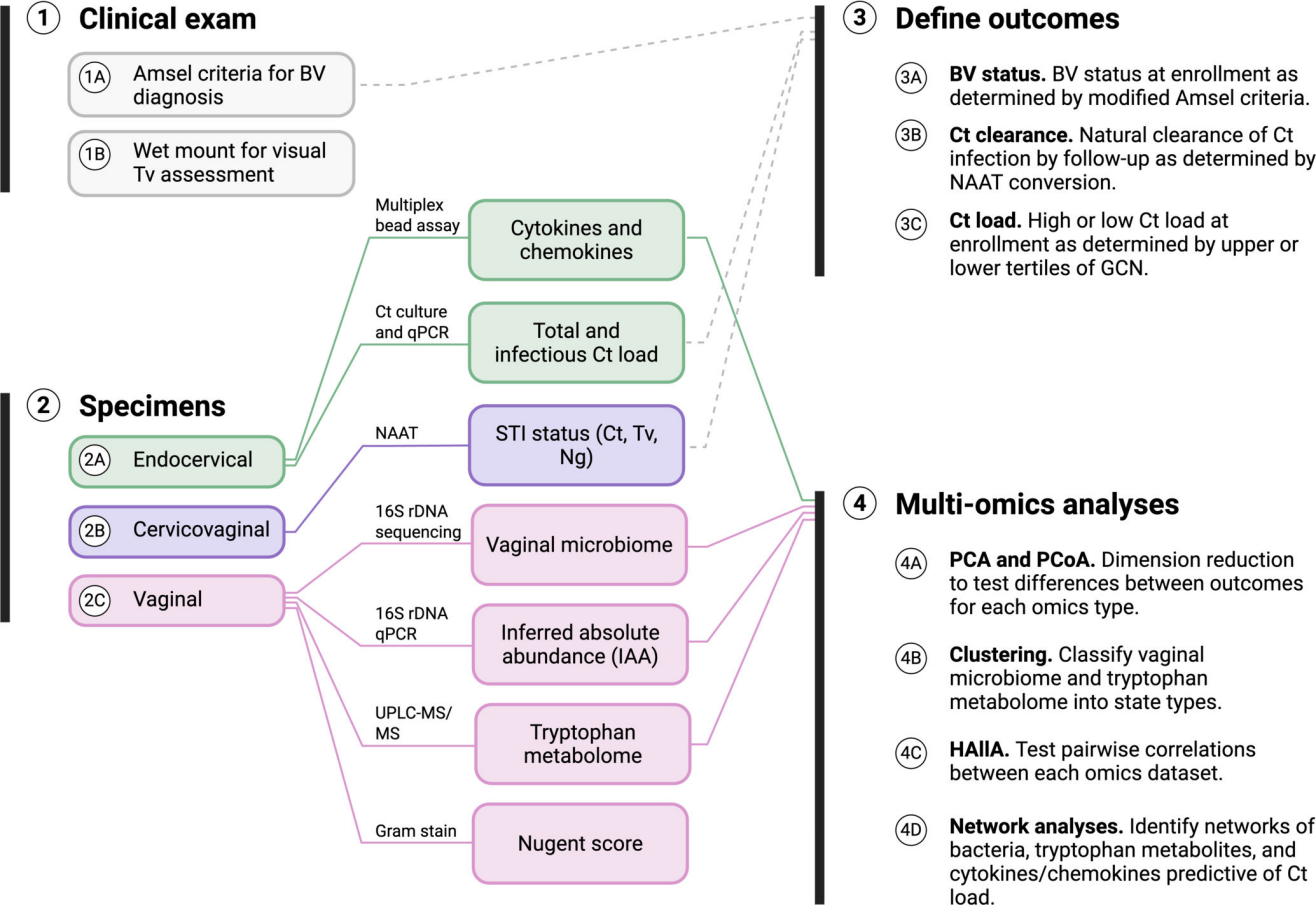
Overview of clinical sampling, assays, and multi-omics analysis. (1) Participants underwent a clinical exam at which they were screened for bacterial vaginosis (BV) and provided cervical and vaginal specimens. (2) Specimens were processed for *C. trachomatis* (Ct), *T. vaginalis*, and *N. gonorrhoeae* nucleic acid amplification testing (NAAT), vaginal microbiome profiling, targeted quantification of vaginal tryptophan metabolites, measurement of endocervical cytokines, and quantification of total and infectious endocervical Ct load (genome copy number [GCN] and inclusion-forming units [IFU], respectively). (3) Participants were grouped by BV status using modified Amsel criteria, Ct natural clearance status defined by NAAT conversion (NAAT-positive at enrollment and NAAT-negative at a follow-up visit 1 week later), and Ct load (high or low, defined by upper and lower tertiles of GCN at enrollment). (4) Multi-omics analyses were performed on enrollment specimens, including dimension reduction (PCA and PCoA), clustering of vaginal microbiome and metabolome into state types, pairwise correlation testing (HAllA), and predictive network analyses (MintTea) to identify microbial, metabolic, and immune features associated with Ct load.

We enrolled young women undergoing routine screening for Ct and BV (visit 1, V1) at the LSU CrescentCare Sexual Health Center, as previously described (2). Fifty-one participants tested Ct-positive by nucleic acid amplification test (NAAT) at V1 and returned for a follow-up Ct treatment visit (visit 2, V2) (2). Forty-nine had a complete vaginal microbiome, metabolome, and cytokine data set and were included in our analyses. An additional 59 women who were not Ct NAAT-positive at V1 and had a complete multi-omics data set were also included in relevant analyses to distinguish BV-related effects from those associated with Ct load (Fig. 1; Table 1).

**TABLE 1:** Characteristics of study participants*^f^*

Characteristic	Total	(*n* = 108)	Ct+	(*n* = 49)	Ct−^*e*^	(*n* = 59)	*P*-value^*a*^
Age, median (range)	23	(18–34)	22	(18–30)	24	(18–34)	0.0054^*b*^
Race, No. (%)							
American Indian	1	(0.93%)	0	(0.00%)	1	(1.69%)	1.0000
Asian	2	(1.85%)	1	(2.04%)	1	(1.69%)	1.0000
Black	81	(75.00%)	43	(87.76%)	38	(64.41%)	0.0070
Hispanic	1	(0.93%)	1	(2.04%)	0	(0.00%)	0.4537
White	22	(20.37%)	4	(8.16%)	18	(30.51%)	0.0042
NA	1	(0.93%)	0	(0.00%)	1	(1.69%)	1.0000
Contraception, No. (%)							
Copper IUD	4	(3.70%)	1	(2.04%)	3	(5.08%)	0.6245
Depo-Provera	2	(1.85%)	0	(0.00%)	2	(3.39%)	0.4997
Hormonal IUD	11	(10.19%)	3	(6.12%)	8	(13.56%)	0.3385
IUD	1	(0.93%)	0	(0.00%)	1	(1.69%)	1.0000
Implant	12	(11.11%)	4	(8.16%)	8	(13.56%)	0.5408
NuvaRing	2	(1.85%)	1	(2.04%)	1	(1.69%)	1.0000
Patch	4	(3.70%)	1	(2.04%)	3	(5.08%)	0.6245
Pills	13	(12.04%)	7	(14.29%)	6	(10.17%)	0.5630
Plan B	2	(1.85%)	0	(0.00%)	2	(3.39%)	0.4997
None	57	(52.78%)	32	(65.31%)	25	(42.37%)	0.0210
STI history, No. (%)	80	(74.07%)	35	(71.43%)	45	(76.27%)	0.6607
Prior *Chlamydia trachomatis*, No. (%)	58	(53.70%)	27	(55.10%)	31	(52.54%)	0.8476
*C. trachomatis* NAAT+, No. (%)							
Cervicovaginal	49	(45.37%)	49	(100.00%)	0	(0.00%)	NA
Oropharyngeal	7	(6.48%)	3	(6.12%)	4	(6.78%)	1.0000
Rectal	5	(4.63%)	4	(8.16%)	1	(1.69%)	0.1742
Coinfections, No. (%)							
Yeast	30	(27.78%)	15	(30.61%)	15	(25.42%)	0.6668
*Neisseria gonorrhoeae*	9	(8.33%)	5	(10.20%)	4	(6.78%)	0.7288
*Trichomonas vaginalis*	8	(7.41%)	5	(10.20%)	3	(5.08%)	0.4641
Metronidazole treatment, No. (%)	77	(71.30%)	38	(77.55%)	39	(66.10%)	0.2076
Nugent category, No. (%)							
0–3	31	(28.70%)	9	(18.37%)	22	(37.29%)	0.0346
4–6	15	(13.89%)	5	(10.20%)	10	(16.95%)	0.4061
7–10	62	(57.41%)	35	(71.43%)	27	(45.76%)	0.0108
Amsel score, No. (%)							
0	10	(9.26%)	0	(0.00%)	10	(16.95%)	0.0018
1	15	(13.89%)	7	(14.29%)	8	(13.56%)	1.0000
2^*c*^	14	(12.96%)	9	(18.37%)	5	(8.47%)	0.1562
Metronidazole Tx	10	(9.26%)	6	(12.24%)	4	(6.78%)	NA
No metronidazole Tx	4	(3.70%)	3	(6.12%)	1	(1.69%)	NA
3^*d*^	29	(26.85%)	15	(30.61%)	14	(23.73%)	0.5142
4	38	(35.19%)	17	(34.69%)	21	(35.59%)	1.0000
NA	2	(1.85%)	1	(2.04%)	1	(1.69%)	1.0000

^*a*^ Fisher’s exact test, unless otherwise indicated.

^*b*^ Wilcoxon rank-sum test.

^*c*^ BV was diagnosed in clinic with modified Amsel’s criteria (Amsel score of 3–4, or Amsel score of 2 and symptomatic) and treated with metronidazole at enrollment (V1).

^*d*^ One participant with an Amsel score of 3 was not treated with metronidazole at enrollment (V1).

^*e*^ CT− includes 52 Ct NAAT-negative, 6 Ct NAAT-inconclusive, and 1 participant without a recorded cervicovaginal NAAT result at enrollment (V1). All specimens in this group were undetectable for Ct by both IFU and GCN assays.

^*f*^ Ct, *Chlamydia trachomatis*; STI, sexually transmitted infection; Tx, treatment; NA, not available/assessed.

BV status was assessed by modified Amsel criteria. All women with a score of ≥3 or with a score of ≥2 with vaginitis at V1, bar one, were treated with metronidazole (MTZ) or Metrogel and were considered BV-positive for this analysis (2). The cohort reflects women at highest risk for Ct infection: the median age was 23 years, 75% identified as Black, 74% self-reported a prior history of a sexually transmitted infection (STI), and 53.7% self-reported a prior history of Ct (25). Vaginal dysbiosis was common at V1, as assessed by modified Amsel criteria (Amsel 4 = 35.19%, Amsel 3 = 26.85%, Amsel 2 with vaginitis = 9.26%) and Nugent scoring (BV = 57.41%, intermediate = 13.89%) (Table 1) (2).

Both infectious (inclusion-forming units, IFU) and total (genome copy number, GCN) Ct loads were quantified in NAAT Ct-positive patients, and, as previously reported, we found a wide spectrum of Ct load at V1 (2). Among women with a positive Ct NAAT result at V1, the upper-tertile median IFU was 41,000 (range, 20,462–845,279), and the lower-tertile median IFU was 1,459 (range, 0–4,524). The upper-tertile median GCN was 10,928,383 (range, 1,692,204–453,843,516), compared with 10,854 (range, 0–75,517) in the lower tertile. The upper and lower GCN tertiles were used to group patients into high and low Ct load groups for analysis (26). Natural clearance occurred between V1 and V2 in approximately 15% of women based on Ct NAAT conversion (NAAT-positive at enrollment and NAAT-negative at a follow-up visit 1 week later) (2).

Vaginal microbiota was profiled by 16S rRNA gene sequencing, and taxon-specific concentrations were inferred using the amplicon sequence variant (ASV)-based inferred absolute abundance (IAA) method (27). Nineteen cytokines and chemokines were quantified from endocervical secretions using a multiplex cytometric bead array as previously described (17). Tryptophan and 26 major tryptophan metabolites were quantified from vaginal wicks using a newly optimized UPLC-MS/MS assay, which had linear standard calibrations up to 10,000 ng/mL and limits of quantitation from 0.9 to 43.3 ng/mL within 20% error, indicating its general suitability for application to biological samples with wide dynamic ranges. Ten metabolites were not detected, had poor reproducibility (likely due to their volatility, e.g., indole), or had a CV of >30%, and were excluded from the analysis. These were 3-hydroxykynurenine, 5-hydroxytryptophan, indole, indole-3-acetaldehyde, indole-3-acetamide, indole-3-pyruvic acid, N-acetylserotonin, serotonin, skatole, and tryptophol.

### Separation of BV and Ct outcomes across microbiome, tryptophan metabolome, and cytokine profiles

**Fig 2:**
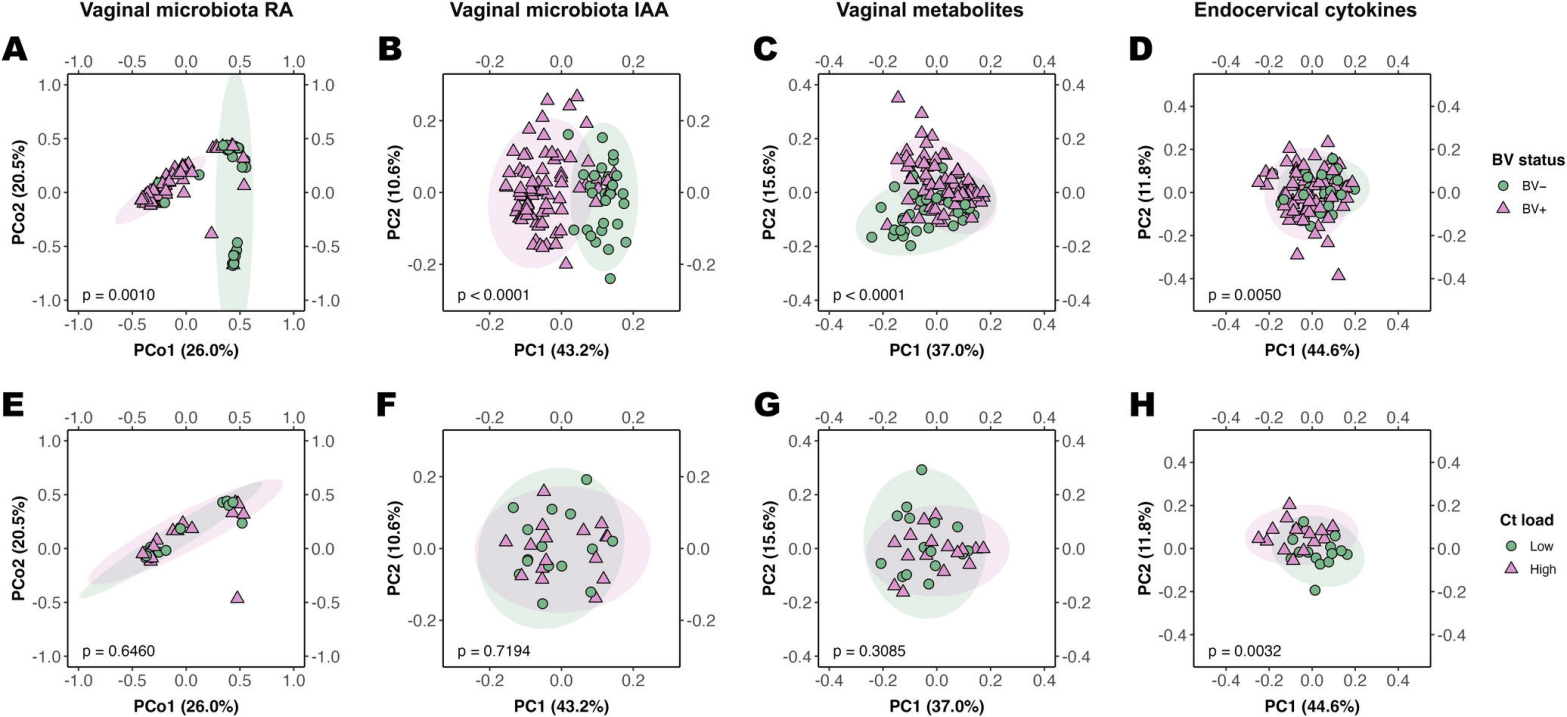
Ordination plots for bacterial abundances, vaginal metabolites, and endocervical cytokines used to separate outcomes. (**A–D**) BV status by modified Amsel criteria. (**E–H**) Ct load (upper versus lower GCN tertiles). (**A and E**) PCoA of ASV relative abundances. (**B and F**) PCA of ASV IAA. (**C and G**) PCA of vaginal metabolites. (**D and H**) PCA of endocervical cytokines. PERMANOVA and MANOVA were used to test for significant separation between groups after PCoA and PCA, respectively.

To characterize broad differences across microbial, tryptophan metabolite, and cytokine profiles, we first performed ordination analyses to determine whether each omics type was separated by BV status, Ct load, or natural clearance of Ct (Fig. 2; Fig. S1). Analyses included principal coordinate analysis (PCoA) of bacterial relative abundance (RA) and principal component analysis (PCA) of bacterial IAA, tryptophan metabolites, and cytokines. Across all omics types, BV status consistently demonstrated significant separation between the BV-positive and BV-negative groups, most significantly by IAA and tryptophan metabolite profiles (Fig. 2A through D). Significant separation between low versus high Ct load, represented using specimen GCN measures, was also observed only with cytokines (Fig. 2E through H). Similarly, Ct natural clearance showed significant separation only by cytokines (Fig. S1). These analyses provide a broad overview of how each omics type distinguishes BV status, Ct load, and Ct natural clearance and establish context for deeper integration of these features.

### Metabolome state types reveal distinct tryptophan metabolism linked to vaginal community composition

**Fig 3:**
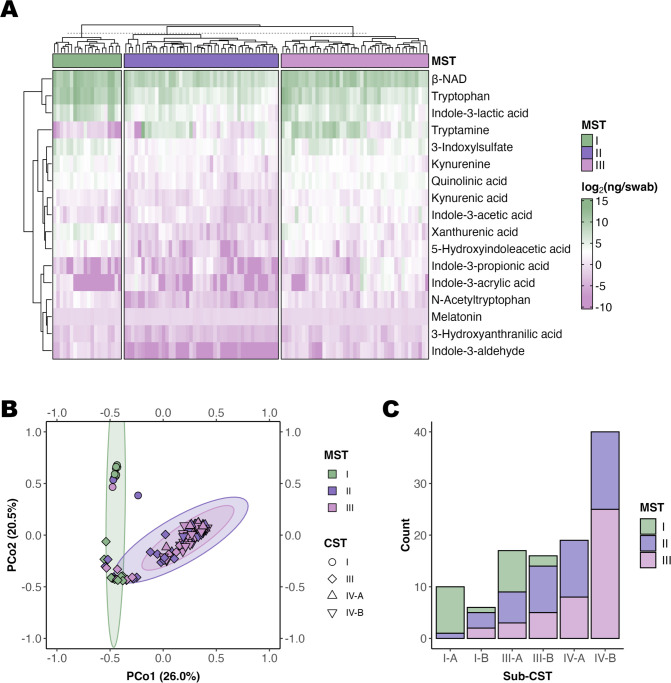
Distinct metabolome state types relate to community state types. (**A**) Heatmap of vaginal metabolite concentrations clustered using K-means into three unique metabolome state types (MSTs). (**B**) PCA of ASV relative abundances. Shapes indicate community state types (CST) assigned using VALENCIA. Colors indicate MST, with 95% confidence ellipses shown. (**C**) Stacked bar plot visualizing the number of specimens classified into each of the MSTs within each VALENCIA sub-CST.

Since tryptophan is central to both BV-associated metabolism and Ct infection, we next examined whether vaginal tryptophan and tryptophan metabolite profiles clustered into discrete metabolic states and how these states relate to vaginal community state types (28). To accomplish this, we applied K-means clustering to metabolite concentrations and identified three metabolome state types (MST) with distinct profiles (Fig. 3A). Differential analysis revealed that tryptophan, indole-3-lactic acid, and xanthurenic acid were significantly enriched in MST I compared to MST II and MST III, while the BV-associated metabolite tryptamine was significantly decreased (Fig. S2). MST II was characterized by a depletion in nearly all metabolites quantified, apart from tryptamine, compared to MST I and MST III (Fig. S2). MST III was characterized by a significant enrichment in multiple downstream indole pathway metabolites, including indole-3-acrylic acid, indole-3-propionic acid, and indole-3-acetic acid, as well as tryptamine and kynurenic acid compared to MST I and MST II (Fig. S2).

We next examined how these metabolic states correspond to vaginal community state types (CST) (28). Overlaying MST assignments onto a PCoA of ASV-level bacterial composition showed that MST II and MST III largely overlapped with the BV-associated CST IV, whereas MST I was more distinct and overlapped with both *Lactobacillus crispatus*-dominant CST I and *Lactobacillus iners*-dominant CST III (Fig. 3B). Metabolomes in MST I had higher odds of being associated with CST I or CST III compared to CST IV-A and CST IV-B, whereas MST II and MST III had higher odds of being associated with CST IV-A and CST IV-B (Data Set S1). CST distributions did not differ between MST II and MST III (Data Set S1). MSTs were further stratified when comparing the relationship between MSTs and *Lactobacillus*-dominant sub-CSTs (Fig. 3C; Data Set S1). Ninety percent of samples in CST I-A and 47% of samples in CST III-A were grouped in MST I. In contrast, samples grouped in MST I made up the smallest portion of the higher-diversity CST I-B (16%) and CST III-B (12.5%). Pairwise Fisher’s exact tests showed that MST I had a significantly different relationship to sub-CSTs compared to MST II and MST III (FDR < 0.001), while MST II and MST III samples did not significantly differ in their relationship to sub-CSTs (FDR > 0.1). Standardized Pearson residuals were calculated to evaluate which sub-CST and MST combinations contributed to observed differences in MST. Based on residual values, MST I samples were overrepresented in CST I-A (residual = 6.1) and CST III-A (residual = 3.3), while MST II (residual = −2.1) and MST III (residual = −2.7) were underrepresented in CST I-A. Additionally, samples in MST III were overrepresented in CST IV-B (residual = 3.6), while MST I samples were underrepresented in CST IV-A (residual = −2.28) and CST IV-B (residual = −3.79) (Data Set S1).

Taken together, these results demonstrate that vaginal tryptophan metabolism segregates into discrete MSTs that closely correspond to microbial community structure. CST I is characterized by high tryptophan availability and kynurenine pathway metabolites (MST I), whereas BV-associated CST IV-A and CST IV-B correspond to metabolomes depleted of tryptophan and enriched in downstream indole pathway metabolites (MST II and MST III). CST III displays intermediate metabolic profiles consistent with its unstable composition.

### Interconnections between the microbiome, tryptophan metabolome, and cytokines

**Fig 4:**
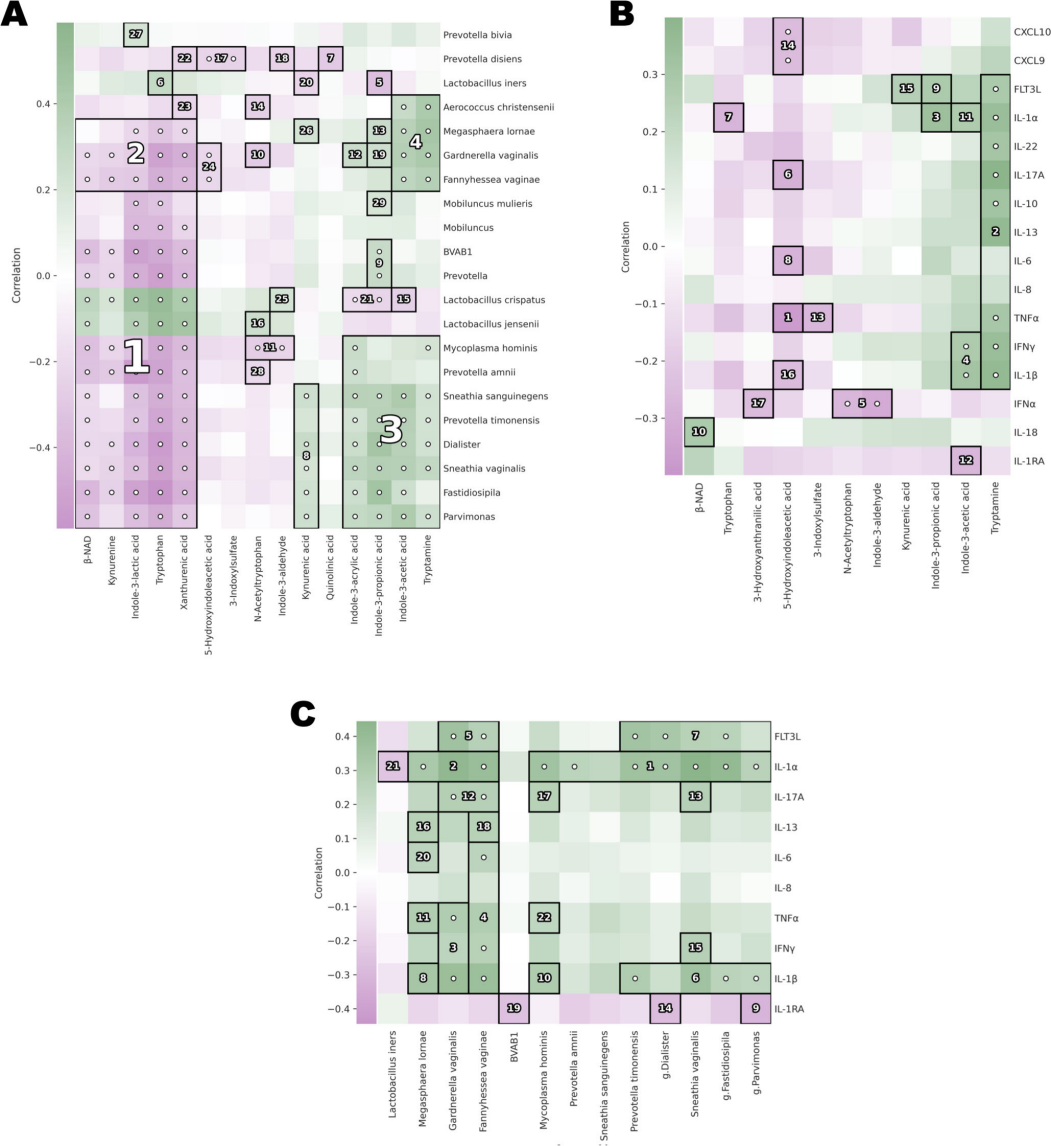
The vaginal microbiome, tryptophan metabolome, and cytokine profiles share interrelated features. The tool HAllA was used to generate associations between metabolites, cytokines, and bacterial taxa. Heatmaps display the Spearman correlation between abundance of (**A**) TSS-normalized taxa and metabolites, (**B**) vaginal immune mediators and metabolites, and (**C**) TSS-normalized taxa and immune mediators. Significant correlations are marked by a dot, and significant clusters of correlations are outlined and numbered. Colors indicate the strength of the correlation between microbial taxa and metabolites. The color scale ranges from pink (negative association) to green (positive association). Hierarchical clustering reveals groups of microbes, cytokines, and metabolites with similar association patterns, highlighting both strong positive and negative associations.

Since MSTs and CSTs showed strong correspondence, we next examined how specific features of each omics type relate to one another by performing hierarchical all-against-all association testing (HAllA) to identify significant relationships among individual features and clusters of vaginal bacterial taxa, tryptophan metabolites, and cytokines (Fig. 4; Fig. S3). Across the microbiome-metabolome matrix (Fig. 4A), HAllA identified 29 clusters composed of 142 significant correlations (FDR < 0.1). *L. crispatus* and *Lactobacillus jensenii*, both considered optimal *Lactobacillus* spp. (dominating CST I and CST V, respectively), demonstrated a positive cluster of correlations with tryptophan, several metabolites in the kynurenine pathway (kynurenine, xanthurenic acid, and β-NAD), and indole-3-lactic acid in the indole pathway. *L. crispatus* abundance also correlated with indole-3-aldehyde, a documented indole pathway metabolite of lactobacilli (29), but negatively with other intermediates produced in the final reactions of tryptophan-indole metabolism, specifically indole-3-acrylic acid, indole-3-propionic acid, and indole-3-acetic acid. *L. iners,* the dominant species of the non-optimal CST III, also positively associated with tryptophan but lacked significant correlations with other metabolites associated with *L. crispatus* and *L. jensenii*.

In contrast, several BV-associated bacteria (BVAB), including *Mycoplasma hominis*, *Prevotella amnii*, *Prevotella timonensis*, *Sneathia vaginalis*, *Sneathia sanguinegens*, *Dialister*, *Fastidiosipila*, and *Parvimonas*, positively clustered with tryptamine, indole-3-acrylic acid, indole-3-propionic acid, and indole-3-acetic acid. A similar correlation cluster of BVAB, including *Aerococcus christensenii*, *Megasphaera lornae*, *Gardnerella vaginalis*, and *Fannyhessea vaginae*, positively correlated with tryptamine, indole-3-propionic acid, and indole-3-acetic acid. *Prevotella disiens* showed a distinct pattern of negative associations with xanthurenic and quinolinic acids (kynurenine pathway), 3-indoxyl sulfate (indican), a downstream metabolite of indole, and indole-3-aldehyde.

HAllA identified 17 clusters composed of 28 significant associations (FDR < 0.1) between tryptophan metabolites and cytokines (Fig. 4B). Tryptophan, which was positively associated with all *Lactobacillus* species, was negatively associated with interleukin (IL)-1α, a cytokine central to BV-mediated inflammation (17). In contrast, tryptamine, which was exclusively associated with numerous BVAB, is positively clustered with numerous proinflammatory cytokines, including IL-1α, IL-1β, IL-17A, IFNγ, and FMS-like tyrosine kinase 3 ligand (FLT3L). Indole-3-propionic acid and indole-3-acetic acid were positively associated with IL-1α, and indole-3-acetic acid was negatively associated with the IL-1 receptor antagonist (IL-1RA).

Across the microbiome-cytokine matrix (Fig. 4C), 22 clusters comprised of 43 significant associations were identified (FDR < 0.1). Numerous BVAB were positively associated with IL-1α, IL-1β, and FLT3L. The abundance of two key BVAB, *G. vaginalis* and *F. vaginae*, was positively associated with the abundance of numerous additional cytokines, including IFNγ, tumor necrosis factor alpha (TNFα), and IL-17A.

In summary, as we and others have previously documented, BVAB are associated with a pro-inflammatory vaginal environment and tryptophan metabolism (17, 23, 30–33). Furthermore, we have shown that BVAB is associated with numerous indole derivatives, specifically indole-3-propionic acid, indole-3-acetic acid, and tryptamine, which may contribute to the inflammatory milieu linked to BVAB.

### Integrated networks predictive of *Chlamydia* load

To evaluate the relationship between Ct load, vaginal microbiome, tryptophan metabolites, and cytokines, we initially investigated their relationship using univariate linear modeling in samples stratified by Ct load. Differential analysis was performed to assess if our omics data differed in abundance based on the high and low range of both our load measures, IFU and GCN, using linear modeling (Data Set S3). Chemokine (C-X-C motif) ligand 9 (CXCL9), CXCL10, and IL-17A were all found to be positively associated with the high IFU group (FDR < 0.1). Additionally, IFNγ and chemokine (C-C motif) ligand 5 (CCL5) trended toward a positive association with the high IFU group (FDR = 0.126). Metabolites and taxa were not found to be significantly associated with Ct load groups using linear modeling, although indole-3-propionic acid and indole-3-acrylic acid trended toward a negative association with the high IFU group (FDR = 0.124).

**Fig 5:**
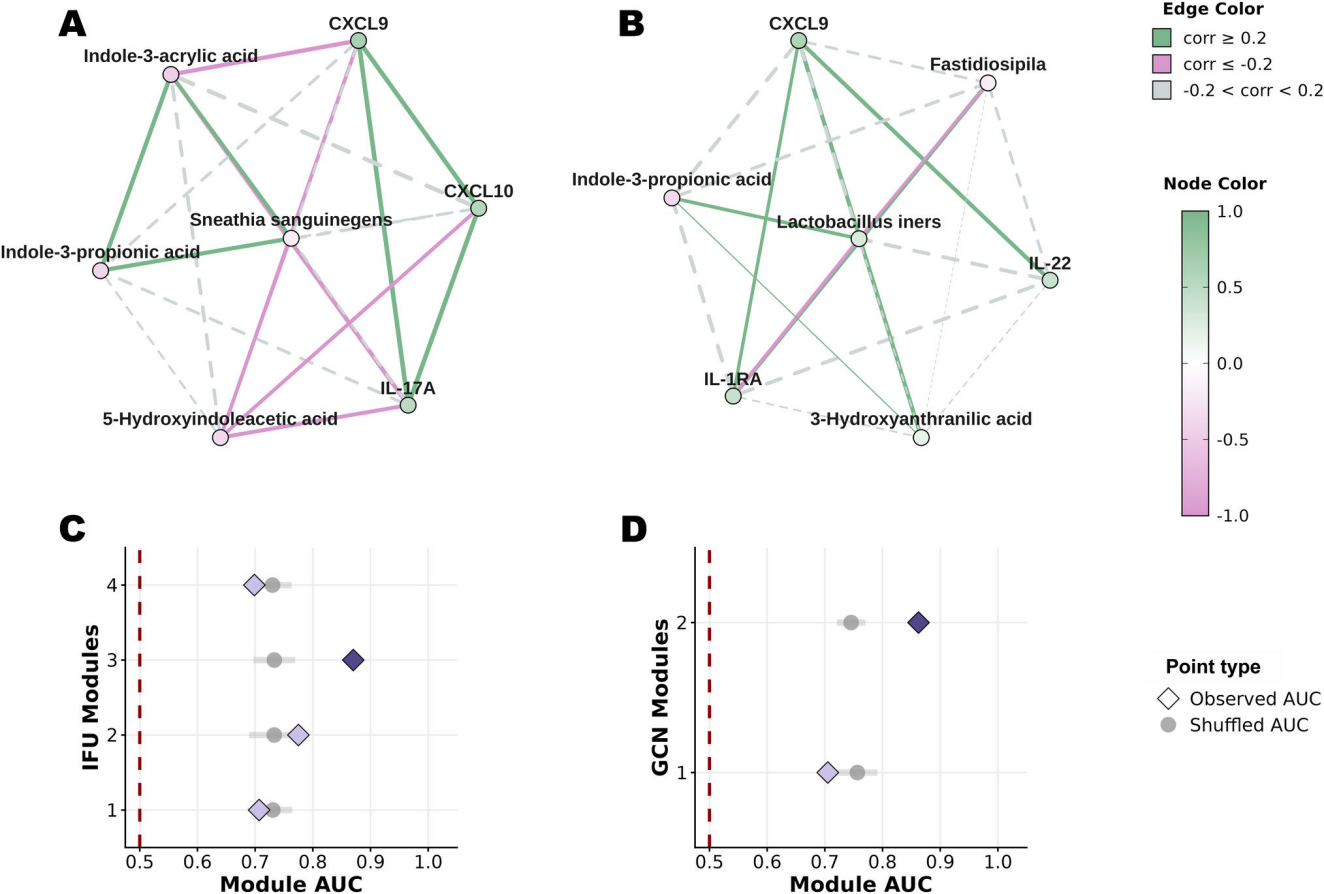
Multi-omic networks differentiate samples based on Ct load. Co-abundance networks were generated using TSS-normalized ASV data, metabolomics data, and cytokine data to separate samples in the high and low Ct load group based (**A**) IFU and (**B**) GCN. Node color indicates the strength of the correlation between the feature and Ct load. The color scale ranges from pink (negative association) to green (positive association). Edge color reflects the correlation between the two features. Gray edge color indicates the correlation coefficient between two features ranges (−0.2, 0.2), green edges indicate a positive correlation with a coefficient ≥ 0.2, and pink edges indicate a negative correlation with a correlation coefficient ≤ −0.2. (**C and D**) Network AUCs were evaluated in comparison to repeated sets of randomly selected (shuffled) features for all co-abundance networks. AUCs of shuffled feature sets are shown in gray, and AUCs from real networks are shown in purple. Mean AUC from co-abundant networks that exceeds the standard deviation of AUC generated from shuffled data is shown in dark purple.

We next used MintTea to identify multi-omic networks predictive of infectious (IFU) and total (GCN) Ct load. Predictive networks were generated using microbial abundance measured with total-sum scaling (TSS)-normalized abundance (Fig. 5) and IAA (Fig. S4). Ct load networks were evaluated in comparison to networks composed of shuffled data, which contain the same number of features as real networks but have randomly selected features. For each of the four experimental conditions, a multi-omics network was found to outperform shuffled networks and achieve robust area under the curve (AUC).

Networks were generated to classify samples with either high IFU (range, 20,462–845,279) or low IFU (range, 0–4,524). Using both microbial abundance metrics, networks classifying participants based on IFU had a robust area under the receiver operating characteristic (AUROC) of 0.89 and 0.87 for networks made from TSS-normalized and IAA data, respectively. Both networks included the BVAB *S. sanguinegens*, indole derivatives indole-3-acrylic acid and indole-3-propionic acid, in addition to the chemokines/cytokines CXCL9, CXCL10, and IL-17A (Fig. 5A; Fig. S4A). Pearson correlation of network features shows that immune mediators CXCL10, CXCL9, and IL-17A are significantly positively associated with IFU (FDR < 0.1).

Additionally, networks were generated to separate samples based on high GCN (range, 1,692,204–453,843,516) and low GCN (range, 0–75,517). Networks that best differentiate samples based on GCN had AUROCs of 0.810 and 0.862 for networks built with TSS-normalized and IAA data, respectively (Fig. 5B; Fig. S4B). GCN networks only shared the inclusion of IL-1RA; however, GCN networks generated with TSS-normalized data included CXCL9 and indole-3-propionic acid, similar to IFU networks. GCN networks generated with IAA additionally included the BVAB *A. christensenii*, the BV-associated metabolite tryptamine, as well as indole-3-acetic acid. Spearman correlation of network features related to GCN found that immune mediators IL-1RA, IL-22, and CXCL9 were positively associated with GCN (FDR < 0.1). Additionally, indole-3-propionic acid was negatively associated with GCN (FDR < 0.1). These findings suggest that key tryptophan metabolites, BVAB, and inflammatory mediators interact with one another, shaping the local cervicovaginal environment and influencing Ct growth and survival. A summary of the results from network analysis is included in Data Set S4.

## DISCUSSION

In this study, we demonstrate three principal findings. First, BV status strongly structures the cervicovaginal microbiome, tryptophan metabolome, and mucosal cytokine environment. Second, variation in cervical Ct load does not align with microbiome composition alone. Third, Ct load most clearly aligns with coordinated microbial-metabolic-immune states defined by specific cytokines and tryptophan metabolites.

A compositional shift to a dysbiotic vaginal microbiome instigates local inflammatory changes and modulates STI infection outcomes in ways we have yet to fully understand (34). BV has been associated with Ct acquisition, persistence, burden type, ascending infection, and risk of reinfection (2, 16, 17, 19, 20, 34). Indole production by BVAB is thought to permit Ct to evade IFN-γ-mediated tryptophan starvation (4, 13, 15). However, we hypothesized that BVAB might also mediate much broader, interconnected changes in tryptophan metabolism and local immunity, which could have significant biological relevance to Ct infection burden. This hypothesis was predominantly inspired by microbial tryptophan metabolism studies in the gastrointestinal tract and their major reported downstream effects on immune regulation and homeostasis (reviewed in references 22–24). We believe that a clearer understanding of the microbiota-*Chlamydia*-host axis in the female genital tract will be required to enhance strategies to characterize, generate, modulate, or preserve the functionality of immunity to Ct, as well as to elucidate the extent and nature of the reservoirs of this chronic infection and associated inflammation.

Unique samples from women enrolled at a Ct and BV screening visit enabled exploration of relationships between the vaginal microbial composition, tryptophan and its metabolites, cervical cytokines, and infectious and total Ct load. Using an integrated omics approach, we aimed to identify ecological networks that related to a BV diagnosis and, importantly, to differences between high versus low cervical Ct load, given that upper tract infection is associated with higher cervical Ct load (3, 4).

Given ours and others’ previous studies broadly characterizing the inflammatory and amino acid-depleted signatures of a vaginal dysbiosis, we were not surprised to observe that the vaginal microbiome, tryptophan metabolome, and cervical cytokine data sets showed clear separation according to a diagnosis of BV (Fig. 2) (17, 30–33). Only cervical cytokines showed separation according to high versus low Ct load. Since our earlier study indicated that a low Ct load was helpful for natural clearance, and high Ct load for evasion of cervical defenses (2), we interpret this to suggest that Ct outcomes may have a distinct endocervical cytokine profile, which was further elucidated in our subsequent analyses.

While we, like others (35), found that the volatility of indole likely precluded it from reliable UPLC-MS/MS detection, our targeted approach enabled a detailed analysis of a major part of the vaginal tryptophan metabolome and its relationship to vaginal bacterial community composition (Fig. 3). We revealed a distinct MST I metabolome predominantly associated with *Lactobacillus* dominance, particularly *L. crispatus* (CST I), which we interpret as a more optimal metabolome for host defense. Here, tryptophan levels approximated those in plasma, indole-3-lactic acid was the predominant indole, and all kynurenine-pathway components were present, including NAD, the final product of tryptophan catabolism via the kynurenine pathway. While the kynurenine pathway only modestly contributes to tryptophan metabolism in mucosal sites, kynurenine is an important feedback mechanism for managing T cell activation and polarization (24, 36). Similarly, kynurenine and many additional metabolites of tryptophan prevent overstimulation of adaptive immunity and mucosal damage (22, 24, 36). Mechanisms include direct cytotoxicity to T cells and a preferential bias toward T regulatory cells and can occur through aryl hydrocarbon receptor (AhR) signaling and IDO1 activation (ibid). NAD is a critical energy source and also maintains mucosal integrity (37).

In contrast to MST I, MST II and MST III were associated with, but could not separate, BV-associated CSTs. Here, tryptophan was significantly depleted; both kynurenine- and serotonin-pathway metabolites were at significantly lower levels than in MST I, and profiles were heavily biased toward downstream indole pathway metabolites generated predominantly by BVAB. MST II was the most severely depleted in tryptophan, but also in many other metabolites. This may reflect greater bacterial consumption in addition to production, or it could reflect a greater level of indole or other metabolites that were not detectable using our methodology. MST III, by contrast, was characterized by a marked enrichment in downstream indole pathway metabolites, including indole-3-acrylic acid, indole-3-propionic acid, and indole-3-acetic acid, as well as elevated kynurenic acid. These metabolites are all well-established AhR and pregnane X receptor (PXR) ligands of varying efficaciousness (38, 39). Further, indole-3-propionic acid and indole-3-acetic acid can induce Ct tryptophan synthase, including under tryptophan sufficiency, which, in the absence of indole, can deaminate serine to pyruvate and ammonia, the latter of which is toxic to Ct (21). Thus, these indoles have important, but complex, implications for Ct infection biology, the outcome of which will be dependent on their combination and concentration in vaginal ecological states *in vivo* (22–24).

Correlations between tryptophan catabolites and vaginal taxa reflect robust differences in the metabolomic profile of BVAB compared to normal vaginal flora. Like previous reports, typical healthy vaginal microbes (viz., *L. crispatus* and *L. jensenii*) were positively correlated with select metabolites, including tryptophan, kynurenine, and indole-3-lactic acid. BVAB showed a strikingly different relationship with tryptophan metabolism (40, 41). Numerous BVAB, such as *G. vaginalis* and *F. vaginae*, were negatively associated with tryptophan abundance and were instead positively associated with downstream indole derivatives, including tryptamine and indole-3-acetic acid. These patterns support a model in which BVAB introduce metabolic capacities absent in *Lactobacillus*, such as tryptophanase and related enzymes that metabolize tryptophan into numerous indole derivatives (42). Consistent with this, BVAB-associated indole derivatives, such as tryptamine and indole-3-acetic acid, were also positively associated with several inflammatory mediators, suggesting coordinated microbial-metabolic-immune interactions. Additionally, *G. vaginalis*, *F. vaginae*, and *M. lornae* were positively associated with numerous inflammatory cytokines. Whether shifts in tryptophan metabolism directly mediate the inflammatory profile associated with BV-associated communities remains unclear, and additional work will be required to dissect these relationships. Our results are particularly notable considering prior work has shown that indole derivatives, such as indole-3-acetic acid and indole-3-propionic acid, maintain epithelial barrier function, regulate T cells, and promote anti-inflammatory responses in the gut. However, similar to our findings, metabolites classically viewed as anti-inflammatory in the gastrointestinal tract (e.g., short-chain fatty acids) can potentiate proinflammatory responses (e.g., TNFα) in cervicovaginal epithelial models (43). Together, these findings suggest that immunoregulatory metabolites in the gut may have more nuanced, context-dependent, and potentially divergent functions in the cervicovaginal environment.

Network analysis aided in further elucidating the relationship between Ct load and the local cervicovaginal environment. Networks that classified samples into high and low Ct load groups shared several targets, including the immune mediators IL-17A, CXCL9, and CXCL10; the indole derivatives indole-3-propionic acid and indole-3-acetic acid; and the BVAB *S. sanguinegens*. These integrated microbial-metabolic-immune modules suggest that Ct load reflects the coordinated influence of specific cytokines, BV-associated taxa, and downstream tryptophan metabolites rather than any single feature alone. However, additional mechanistic studies will be required to clarify how these microbial and immune pathways converge to shape Ct growth and persistence *in vivo*.

Our finding that high Ct load is positively associated with cervical CXCL9, CXCL10, and IL-17A is intriguing and important. These cytokines have previously been associated with detection of Ct in the upper female reproductive tract, together with TNFα, CCL4, CXCL11, and CXCL13, the latter two of which were not included in our panel (4). Taken together, these studies strongly suggest that elevated CXCL9, CXCL10, and IL-17A mark higher burden infections that have been associated with increased risk of upper reproductive tract involvement and adverse reproductive outcomes. Both also support the interpretation that local host immunity is modulated by the microbiome rather than solely reflecting Ct load. Interestingly, ours and others’ earlier *in vitro* studies demonstrated that Ct infection abrogates cervical epithelial cell CXCL10 secretion (44–46). Further, CXCL10 is generally decreased in the vaginal secretions from BV patients (17, 47, 48). How might these *in vitro* observations reconcile with those *in vivo*? First, our study quantifies cytokines produced by the cervix, the primary site of Ct infection, rather than those derived from the vagina. Second, it is possible that the early, innate epithelial response to Ct, initiated by IL-1α (49), induces inflammation and amplifies the immune response, including chemokine-producing macrophages and dendritic cells (50). As in the murine model of Ct, this may draw in “bystander” CXCR3+ T cells (i.e., T cells with receptors for CXCL9 and CXCL10) that are not Ct-specific and do not contribute to Ct clearance but likely contribute to immunopathology (51). In the highly fluctuating ecological state of the human female genital tract, where Ct can adapt, survive, and establish chronic infection, local levels of these chemokines would therefore be expected to increase with Ct load. Similarly, IL-17-producing cells, which can contribute to a robust generation of Th1 immunity (52, 53), can also later induce tissue damage, primarily via amplifying inflammation through matrix metalloproteinase-9 (MMP9) induction and neutrophil infiltration (reviewed in reference 54), the latter of which is associated with human cervical Ct infection (55).

The antagonism of BV to the natural resolution of Ct infection has been documented through a longitudinal natural history study (20) and by the impact of successful metronidazole treatment of BV (17), in addition to *in vitro* studies with indole (4, 13, 15). However, many conundrums remain (34). Indole cannot be reliably quantified from genital samples from Ct-positive women, likely due to its volatility. Additionally, vaginal amino acid depletion, a hallmark of dysbiosis, has recently been associated with natural clearance of Ct (56). An optimal *Lactobacillus*-dominant environment, despite the highest tryptophan levels, aids clearance and low Ct load (2, 17, 20), likely due to multiple *L. crispatus*-synthesized factors (43, 57). Further, while multiple studies illuminate the role of a polymicrobial microbiota and its metabolic products in shaping and maintaining mucosal immunity, these are all focused on the gastrointestinal (GI) tract. In contrast to the GI tract, the healthy lower female genital tract is dominated by *L. crispatus* or *L. jensenii*, there are no organized genital immune inductive sites, and a polymicrobial microbiome is considered dysbiotic and inflammatory (58). How do we reconcile these disparate findings? We previously hypothesized that Ct may be able to survive, and perhaps even thrive long-term, in a mildly dysbiotic environment we think of as a “Goldilocks zone” (2). We further refine this hypothesis to suggest that there may be an ecological niche that includes (i) sufficient, if not optimal, tryptophan that either allows Ct to survive, replicate, and successfully enter and exit from a persistent state permitting the establishment, maintenance, and amplification of infection; (ii) individual and groups of tryptophan metabolites that contribute to chlamydial survival or evasion of immunity, either directly or indirectly; and (iii) a local immune composition that is modified by BVAB and its products and that contributes to inflammation, but not necessarily clearance. An attractive, alternative, and non-exclusive interpretation is that the mucosal Ct genotypes (D–K) have become broadly adapted to tolerate a wide range of tryptophan and tryptophan metabolite concentrations encountered at urogenital and gastrointestinal mucosal surfaces *in vivo*, shifting metabolic strategies in response to local nutrient availability and host defenses rather than being strictly constrained by them. This interpretation is consistent with the distinct disease phenotypes and tissue tropism of these mucosal genotypes compared with the invasive lymphogranuloma venereum (L1–L3) and ocular-tropic strains (A–C) (21, 59, 60). Such metabolic flexibility may also help explain the ability of Ct to persist over extended periods despite ecological fluctuations in the genital tract, including microbiome shifts associated with bacterial vaginosis and physiological changes related to menstruation or sexual activity (61).

There are a number of limitations to this study. The cohort size is modest, and species-level analyses are constrained by 16S rRNA gene sequencing. While we have previously documented persistent Ct forms in the cervix (62) and investigated their adaptation to varying tryptophan levels (63), IFU and GCN cannot fully distinguish these forms, which are likely to provide critical information on Ct adaptation and survival *in vivo* and how they contribute to chronic infection and inflammation. As with nearly all human Ct studies, the duration of participants’ Ct infection was unknown, which may have confounded some measures, such as cytokines. Interpretation of optimal *Lactobacillus*-dominant microbiomes was also constrained by relatively smaller group sizes compared to BV-associated CST IV, limiting power to detect load-associated differences within *Lactobacillus*-dominant states, especially for the *L. crispatus*-dominant CST I.

In conclusion, this study provides the first quantitative analysis of tryptophan and its metabolites in optimal and non-optimal vaginal environments and supports the concept that Ct infection outcomes are shaped by coordinated microbial-metabolic-immune interactions rather than any single factor. Importantly, although BVAB were incorporated within modules predictive of Ct load, variation in cervical Ct load aligned most clearly with integrated microbial-metabolic-immune states in which cytokine and tryptophan metabolite features most clearly separated high and low Ct load. These findings refine current models of Ct-BV interactions by suggesting that microbial effects on Ct load are mediated through coordinated metabolic and immune states rather than microbiome composition alone. By identifying multi-omic modules associated with high versus low Ct load, this work provides a framework for future studies aimed at defining pathways that may influence persistence, clearance, and potential ascension of Ct infection.

## MATERIALS AND METHODS

### Study population and sample collection

This research was approved by the Institutional Review Board of Louisiana State University Health Sciences Center, New Orleans (Protocol no. 1081). All participants provided written informed consent, and all research was undertaken in accordance with the federal guidelines. The study design, enrollment criteria, and sample collection and processing protocols have been described previously (2). In brief, women (18–35 years old) were recruited from the LSU CrescentCare Sexual Health Clinic in New Orleans, and samples were taken at a Ct screening visit (visit 1, V1) and, if Ct NAAT-positive, approximately 1 week later at a follow-up visit for Ct treatment (visit 2, V2). Samples from 59 women who were not Ct NAAT-positive at V1 (52 Ct NAAT-negative, 6 Ct NAAT-inconclusive, and 1 with a missing NAAT entry) and who had a similar prior history of Ct were also included in the study.

Samples were collected and analyzed as previously described (2, 17). Endocervical secretions were sampled with (i) a Dacron swab immediately immersed in 1 mL sucrose-phosphate-glutamate (SPG) buffer-based Ct transport medium (Ct genomes and IFU) and (ii) absorption with a Merocel sponge (cytokines). The cervicovaginal region was sampled using an endocervical swab with a vaginal drag (NAATs for Ct, *Neisseria gonorrhoeae* [Ng], and *Trichomonas vaginalis* [Tv]). Vaginal secretions were sampled with (i) a Weck-Cel sponge (metabolome), (ii) a Copan swab immediately placed in 1 mL AssayAssure Genelock (Sierra Molecular) (microbiome), (iii) a cotton swab for assessing Amsel criteria, and (iv) a cotton swab used for slide preparation for Gram staining and Nugent scoring. Genital research samples were immediately placed on ice after collection, processed within 2 h, after which they were stored at −80°C until analysis.

### STI testing and Ct load quantification

Ct and Ng were detected by the Aptima Combo 2 test (Hologic, Marlborough, Massachusetts), and Tv was detected by the Aptima Tv assay (Hologic, Marlborough, Massachusetts). BV was diagnosed in the clinic by Amsel criteria and later evaluated by Nugent scoring as an additional, non-identical BV measure as previously reported in Ardizzone et al. (2, 64, 65). Tv was also assessed by wet mount microscopy at the time of clinical evaluation, with immediate treatment initiated if organisms were observed. Women with an Amsel score of 3–4 or symptomatic (odor, discharge, or irritation/itching) with an Amsel score of 2 (modified Amsel criteria) were treated with metronidazole (MTZ) at V1 (2, 66). Women who tested positive by Ct NAAT at V1 were treated with azithromycin or doxycycline at V2, and those with a positive Ng NAAT at V1 were treated with ceftriaxone at V2. Women who developed BV or failed to resolve BV in the interim between V1 and V2 were given MTZ at V2. Ct load was determined by IFU and GCN, which were quantified in parallel from the same endocervical swab as previously described (2). Prior to analysis, values were log_10_-normalized.

### Vaginal swab DNA isolation and 16S rRNA gene sequencing

As previously described, DNA was extracted from vaginal Copan swabs and processed for 16S rRNA gene sequencing with appropriate controls at the Indiana University Center for Genomics and Bioinformatics (2).

### Inferred absolute abundance of bacterial taxa

The estimated abundance of vaginal bacterial concentrations, the inferred absolute abundance (IAA), was calculated from the total bacterial load (27). Total bacterial loads in vaginal Copan swabs were determined by quantitative PCR (qPCR) using methods described previously (67). The resulting IAA was log_10_-transformed.

### Clustering into community state types and metabolome state types

K-means clustering was used to generate three unique metabolome state types (MST), and VALENCIA was used to generate the community state types (CST) (28); CST I is *L. crispatus*-dominant, CST III is *L. iners*-dominant, and CST IV is BVAB-dominant. Principal coordinate analysis was used to observe the relationship between the CSTs and MSTs. Fisher’s exact test was used to evaluate if there is a significant association between CSTs and MSTs, as well as sub-CSTs and MSTs. Chi-squared tests were used to calculate standardized residuals. Standardized residual values were used to assess which MST and CST/sub-CST combinations were over- or underrepresented in the cohort. Additionally, odds ratios were calculated for MST-CST combinations to evaluate the odds of a specific MST-CST combination occurring. Maaslin2 was used to evaluate metabolites that differed significantly based on MST.

### Targeted tryptophan metabolomics

Tryptophan metabolites from serum and vaginal secretions were quantified using an optimized ultra-performance liquid chromatography-tandem mass spectrometry (UPLC-MS/MS) assay (68). Tryptophan, quinolinic acid, picolinic acid, xanthurenic acid, kynurenic acid, kynurenine, 3-hydroxykynurenine, NAD, N-acetyltryptophan, N-acetylserotonin, tryptamine, serotonin, skatole, indole, indole-3-propionic acid, indole-3-aldehyde, indole-3-acetaldehyde, indole-3-pyruvic acid, indole-3-acrylic acid, indole-3-acetamide, tryptophol, 3-hydroxyanthranilic acid, and melatonin were purchased from Sigma Aldrich. Indole-3-acetic acid and indole-3-lactic acid were from Combi-Blocks, Inc.; 5-hydroxytryptophan from Alfa Aesar; 5-hydroxyindoleacetic acid from Tokyo Chemical Industry; and 3-indoxyl sulfate from Toronto Research Chemicals. All isotopically labeled compounds used as internal standards (ISTD) were obtained from Cambridge Isotope Laboratories. All reagents used were mass spectrometry grade and obtained from Fisher Scientific. All stock standards (1 mg/mL) were prepared in ethanol or water, according to the manufacturer’s instructions. Working calibration standards were 0–10,000 ng/mL.

Each vaginal Weck-cel sponge was added to 500 µL of 80% methanol spiked with ISTD in a 1.5 mL tube at 4°C for 30 min. The sponge was squeezed against the tube wall, placed inside a Costar Spin-X 0.22 µm centrifuge tube filter cup (Corning), cut to fit, and then the filter cup was inserted into the 1.5 mL tube of solvent and spun at 18,000 RCF for 10 min. Another 500 µL of 80% methanol spiked with ISTD was added to the filter cup with the Weck-cel sponge and incubated at 4°C for 30 min. The filter tube assembly was spun again at 18,000 RCF for 10 min. The filter cup and Weck-cel sponges were removed before the combined extracts (1 mL) were evaporated by SpeedVac. Dried extracts were resuspended in 100 µL of 50% methanol before transferring to a 96-well plate, and then, 10 µL of each sample was combined to make a QC pool. Both QC samples were used to condition the LC/MS systems and were also injected between every 12 analytical samples. The QC data were also used to evaluate the reproducibility of each assay.

Chromatographic separation was performed on an ACQUITY Premier UPLC (Waters Corporation) fitted with an HSS T3 C18 reverse-phase column (2.1 × 100 mm, 1.8 µm) kept at 50°C, while samples were kept at 10°C. A flow rate of 500 µL/min and an injection volume of 4 µL were used. Mobile phases consisted of 0.1% formic acid in 10 mM ammonium formate (A) and 0.1% formic acid in methanol (B), with a 12-minute elution gradient as follows: hold 0% B for 1 min, ramp to 30% B over 4 min, ramp to 100% B over another 4 min, hold 100% B for 2 min, return to 0% B over 6 s, and then hold 0% B until 12 min is reached. Quantification was carried out on a Xevo TQ-S Micro Triple Quadrupole Mass Spectrometer (Waters Corporation), with data acquisition and analysis performed using MassLynx V4.2 and TargetLynx XS software, respectively. Multiple reaction monitoring (MRM) data were acquired in positive and negative electrospray ionization modes with parameters shown in Table S1.

Ten metabolites were excluded from the analysis as they were not detected, had poor reproducibility (likely due to their volatility), or had a CV >30% (3-hydroxykynurenine, 5-hydroxytryptophan, indole, indole-3-acetaldehyde, indole-3-acetamide, indole-3-pyruvic acid, N-acetylserotonin, serotonin, skatole, and tryptophol) (Table S1). Concentrations of the remaining metabolites were expressed as ng/swab and log_2_-transformed.

### Quantification of cervical cytokines

Cervical secretions were eluted from Merocel sponges in a spin assembly apparatus as previously described (69). IFNα, IFNγ, IL-1α, IL-1β, IL-6, IL-10, IL-12, IL-13, IL-17A, IL-18, IL-22, CXCL8, CXCL9, CXCL10, CCL3 (MIP-1α), CCL4 (MIP-1β), CCL5, FLT3L, and TNFα were quantified by a cytometric bead array assay (MILLIPLEX MAP Immunology Multiplex Assay, Millipore), following the manufacturer’s instructions and as previously described (17). Cytokine measurements below the limit of detection were assigned to a value of half of the minimum detectable concentration for that cytokine. CCL3 and CCL4 were not detected in at least 50% women and were excluded from downstream analyses.

### HAllA analysis

HAllA (v0.8.20) was used to find associations between vaginal bacteria, tryptophan metabolites, and cytokines (70). HAllA is an end-to-end statistical method for hierarchical all-against-all discovery of significant relationships among data features with high power. Significant relationships were denoted with a white dot, and block associations denoted with numbered blocks.

### MintTea network analysis of networks predictive of Ct load

Microbiome, metabolites, and cytokine data were integrated into networks of related features predictive of Ct load using the R package MintTea (v1.0.0) (71). The samples that tested positive for Ct by NAAT were subset into equal thirds (tertiles) of high, medium, and low levels of Ct load. This grouping was performed using GCN and repeated using IFU so that each metric could be evaluated independently as a measure of Ct load. Samples in the upper and lower tertiles were retained for analysis with MintTea. Analysis was performed separately for participants grouped based on GCN and IFU. Parameters for each MintTea analysis were optimized using a grid search approach to aid in identifying networks most predictive of Ct load. Parameters optimized included edge threshold, number of feature constraints, and data folds used for sGCCA analysis. Final network parameters were selected based on which parameters produced the module with the highest AUROC and an inter-omic correlation ≥0.2. AUROC values were generated using 5-fold cross-validation with 20 repeats. Separate networks were generated using TSS-normalized taxonomic abundance and IAA. Additionally, networks were generated separately for Ct load measured by IFU and GCN. Spearman correlation was used to assess the relationship between network nodes and to assess the relationship between Ct abundance measures and network nodes. Maaslin2 was used to perform linear modeling that evaluated whether omics features differed in abundance based on high or low Ct load category using IFU and GCN independently.

## Data Availability

The data sets presented in this study can be found under BioProject PRJNA1013329.

## SUPPLEMENTAL MATERIAL

Data S1 iai.00681-25-s0001.xlsx MST metabolite abundance comparisons & MST vs. CST statistical testing.

Data S2 iai.00681-25-s0002.xlsx Pearson correlations between omics data.

Data S3 iai.00681-25-s0003.xlsx Linear modeling comparing omics feature abundance in participants stratified by Ct load.

Data S4 iai.00681-25-s0004.xlsx Inter-omic network summary and feature correlations.

Fig. S1 iai.00681-25-s0005.tiff Ordination of multi-omics data based on Ct natural clearance status.

Fig. S2 iai.00681-25-s0006.tif Metabolite abundance differs based on MST.

Fig. S3 iai.00681-25-s0007.tiff Inter-omics correlations between taxonomic data measured using IAA, metabolomics, and cytokine profiles.

Fig. S4 iai.00681-25-s0008.tif Multi-omics networks differentiate samples based on Ct load category.

Supplemental material iai.00681-25-s0009.docx Supplemental figure legends.

Table S1 iai.00681-25-s0010.docx Metabolomic parameters.

ASM does not own the copyrights to Supplemental Material that may be linked to, or accessed through, an article. The authors have granted ASM a non-exclusive, world-wide license to publish the Supplemental Material files. Please contact the corresponding author directly for reuse.
