# Supplementary figures and images for: Integrated multi-omics analysis uncovers cervicovaginal ecological networks and their association with Chlamydia trachomatis load
Source: Infect Immun. 2026 Jun 12;94(7):e00681-25. doi: 10.1128/iai.00681-25 (PMC13367043; doi:10.1128/iai.00681-25)

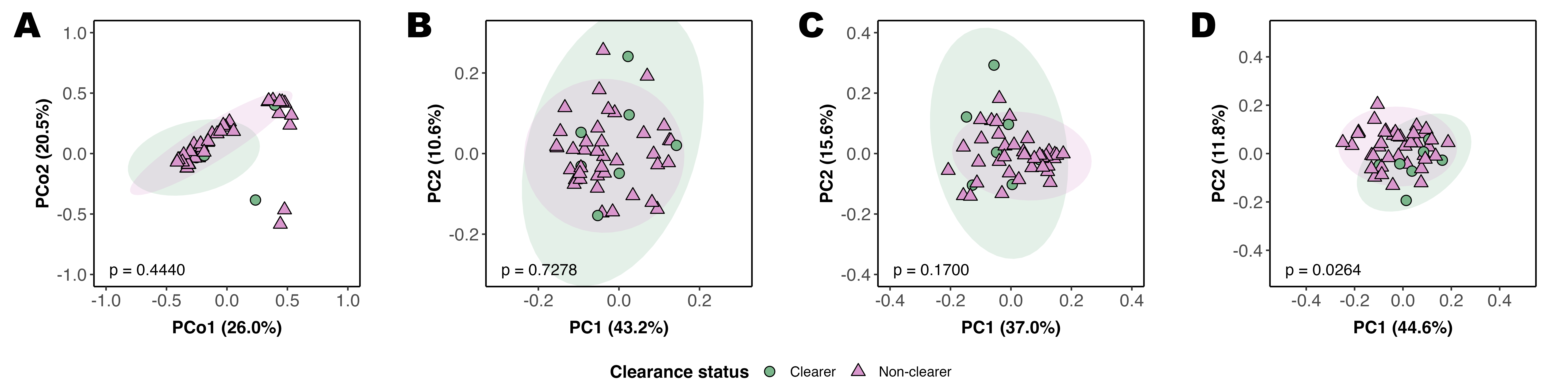

Supplement: Fig. S1 — Ordination of multi-omics data based on Ct natural clearance status. [file iai.00681-25-s0005.tiff]

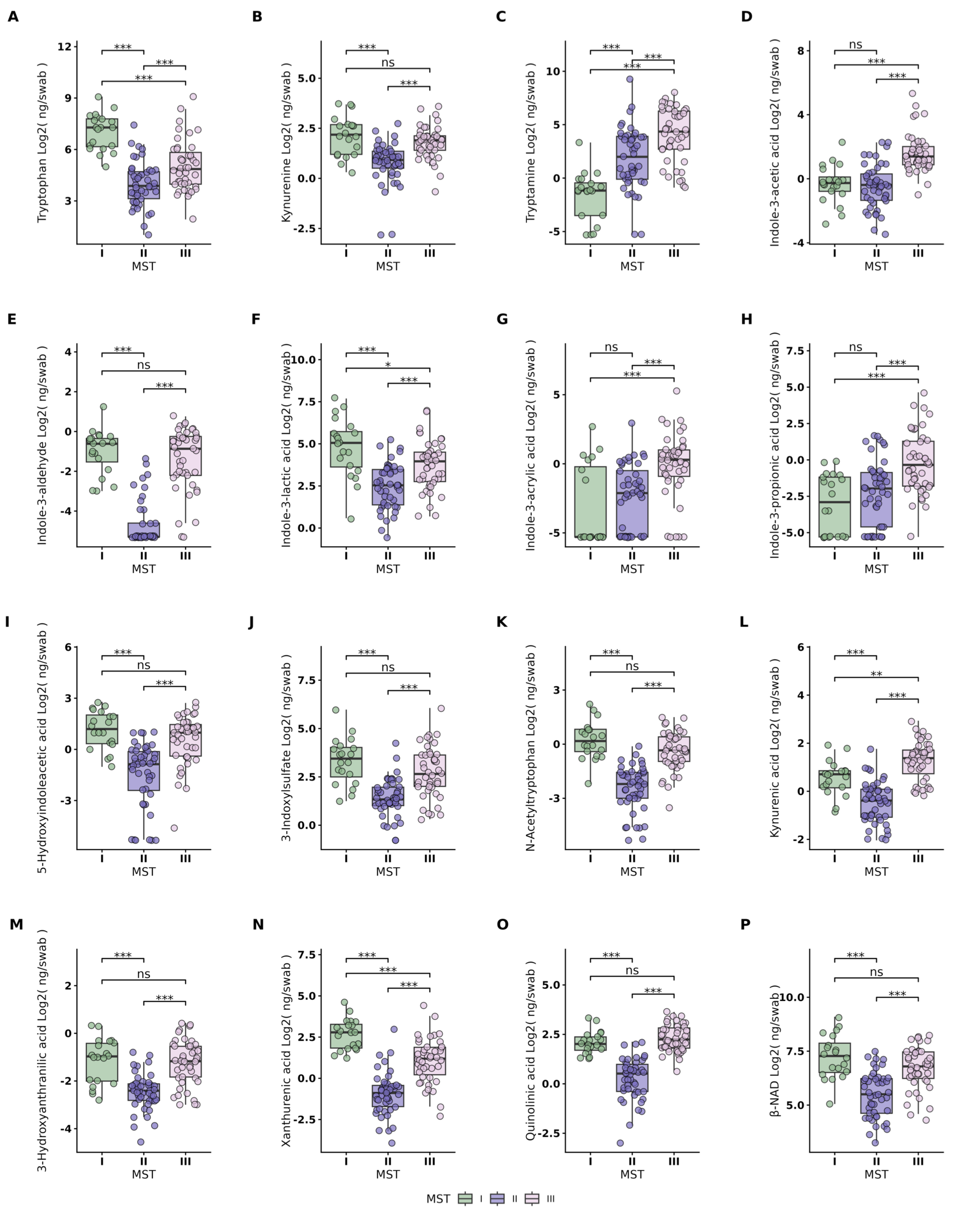

Supplement: Fig. S2 — Metabolite abundance differs based on MST. [file iai.00681-25-s0006.tif]

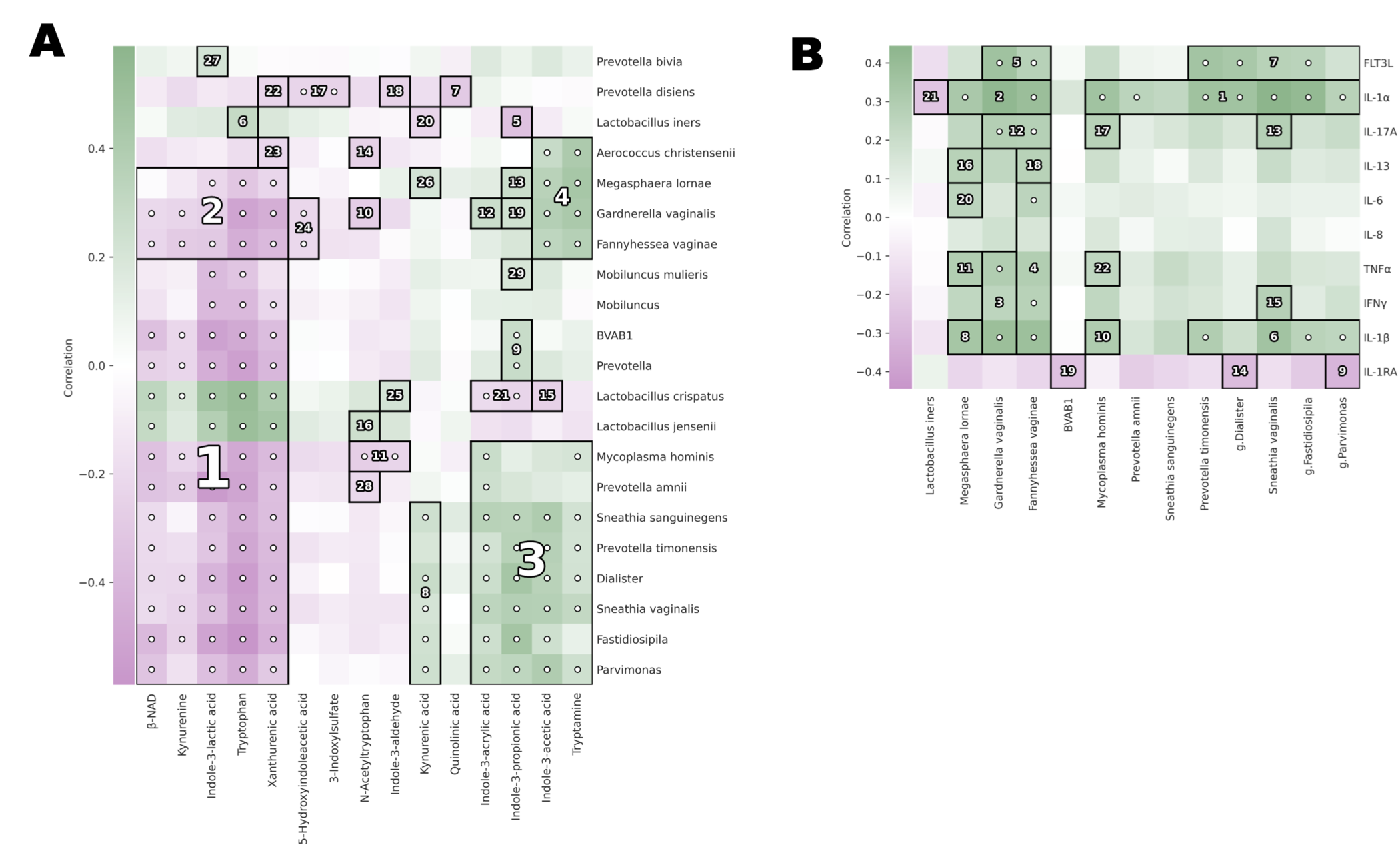

Supplement: Fig. S3 — Inter-omics correlations between taxonomic data measured using IAA, metabolomics, and cytokine profiles. [file iai.00681-25-s0007.tiff]

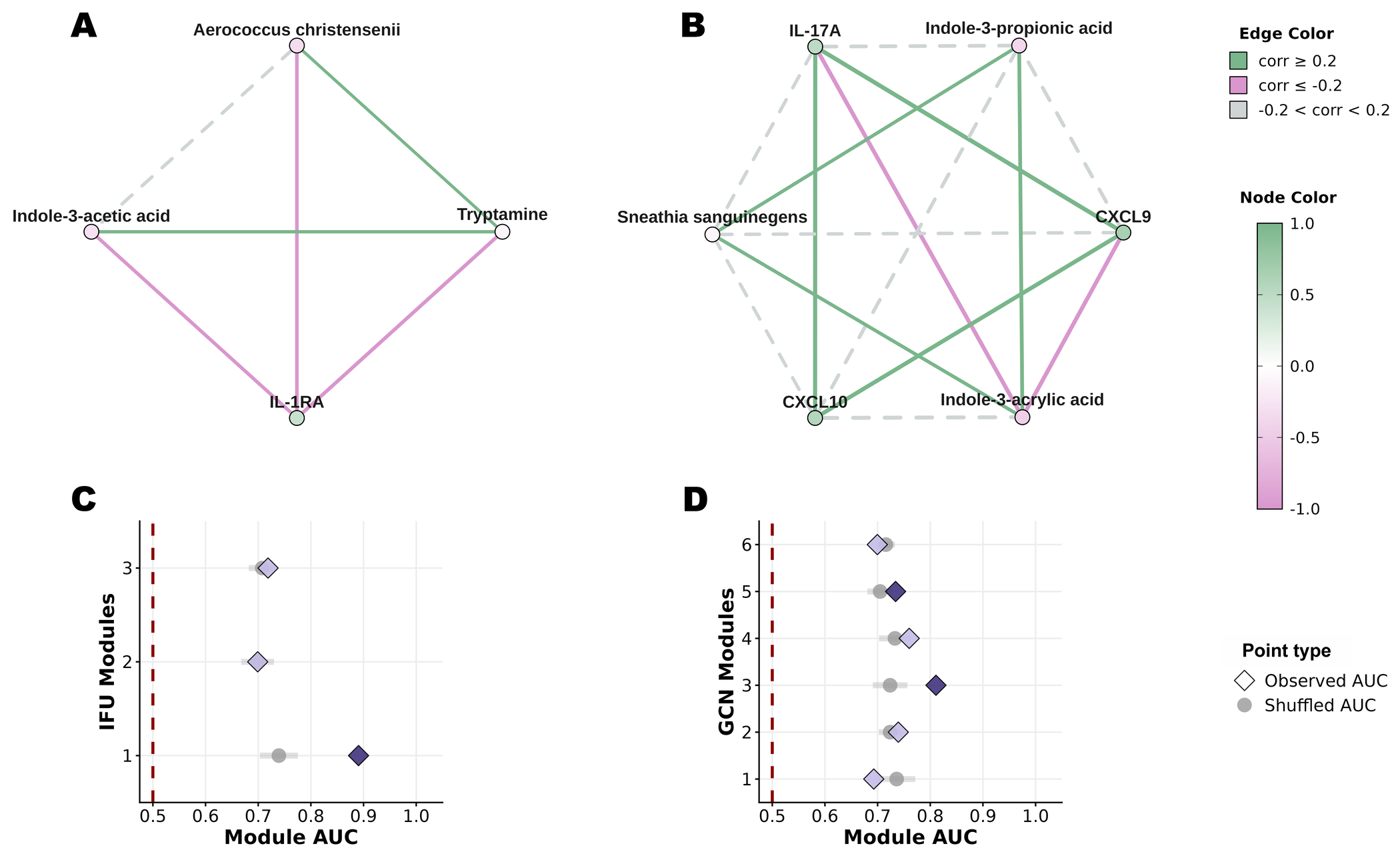

Supplement: Fig. S4 — Multi-omics networks differentiate samples based on Ct load category. [file iai.00681-25-s0008.tif]
