# Supplementary material for: Integrated multi-omics analysis uncovers cervicovaginal ecological networks and their association with Chlamydia trachomatis load
Source: Infect Immun. 2026 Jun 12;94(7):e00681-25. doi: 10.1128/iai.00681-25 (PMC13367043; doi:10.1128/iai.00681-25)
Supplement: Supplemental material — Supplemental figure legends. [file iai.00681-25-s0009.docx]

# SUPPLEMENTAL FIGURE LEGENDS

**Fig. S1. Ordination of multi-omics data based on Ct natural clearance status.** PCoA was used to evaluate if multi-omics data separated based on whether a participant cleared Ct infection between V1 and V2 as determined by NAAT conversion. (**A**) PCoA of ASV relative abundances. (**B**) PCA of ASV IAA. (**C**) PCA of vaginal metabolites. (**D**) PCA of endocervical cytokines. PERMANOVA and MANOVA were used to test for significant separation between groups after PCoA and PCA, respectively.

**Fig. S2.** **Metabolite abundance differs based on MST.** Metabolite abundance measured as log_2_(ng/swab) was compared between MST using Maaslin2. Metabolites that differed significantly between MSTs were plotted in the above figure. Samples grouped in MST I, MST II, and MST III were shown in green, purple and pink respectively. “ns” indicates non-significant difference in abundance (FDR > 0.05), * = (FDR < 0.05), ** = (FDR < 0.01), *** = (FDR < 0.001)

**Fig. S3.** Inter-omics correlations between taxonomic data measured using IAA, metabolomics, and cytokine profiles. (A) HAllA was used to generate inter-omic correlations between bacterial IAA and abundance tryptophan catabolome targets. (B) Similarly, correlations between IAA and cytokines were evaluated. Significant individual correlations are marked by a dot, and significant clusters of correlations are numbered. Colors indicate the strength of the correlation between microbial taxa and metabolites. The color scale ranges from pink (negative association) to green (positive association). Hierarchical clustering reveals groups of microbes, cytokines, and metabolites with similar association patterns, highlighting both strong positive and negative associations.

**Fig. S4.** Multi-omics networks differentiate samples based on Ct load category. Co-abundance networks were generated using bacterial IAA, metabolomics data, and cytokine data to separate samples in the high and low Ct load group based (A) IFU and (B) GCN. Node color indicates the strength of the correlation between the feature and Ct load. Edge color reflects the correlation between the two features. Grey edges indicate a correlation coefficient (-0.2, 0.2), green edges indicate a positive correlation with a coefficient ≥ 0.2, and pink edges indicates a negative with a correlation coefficient ≤ -0.2. (C-D) Network AUCs were evaluated in comparison to repeated sets of randomly selected (shuffled) features for all co-abundance networks that were generated. AUCs of shuffled feature sets are shown in grey, and AUCs from networks are shown in purple. Mean AUCs from co-abundant networks that exceed the standard deviation of AUC generated from shuffled data are shown in dark purple.
