# Supplementary material for: Integrated multi-omics analysis uncovers cervicovaginal ecological networks and their association with Chlamydia trachomatis load
Source: Infect Immun. 2026 Jun 12;94(7):e00681-25. doi: 10.1128/iai.00681-25 (PMC13367043; doi:10.1128/iai.00681-25)
Supplement: Table S1 — Metabolomic parameters. [file iai.00681-25-s0010.docx]

**Table S1. Metabolomics parameters.**

| **Compound** | **Parent (m/z)** | **Quantifier (m/z)** | **Qualifier (m/z)** | **Cone (V)** | **Collision (V)** | **RT (min)** | **LOD (ng/ml)** | **LOQ (ng/ml)** | **ISTD Used** |
| --- | --- | --- | --- | --- | --- | --- | --- | --- | --- |
| Quinolinic Acid | 168.0 | 78.0 | 150.0 | 22 | 18 | 0.83 | 3.3 | 10.9 | Kynurenine-13C10 |
| 3-Hydroxykynurenine | 225.1 | 208.0 | 110.0 | 8 | 8 | 1.20 | 39.4 | 119.5 | Kynurenine-13C10 |
| NAD | 664.1 | 136.0 | 428.0 | 60 | 60 | 1.50 | 138.4 | 419.5 | Kynurenine-13C10 |
| 5-Hydroxytryptophan | 221.1 | 204.0 | 162.0 | 28 | 10 | 2.35 | 8.4 | 28.1 | Tryptophan-d5 |
| Kynurenine-13C10 | 219.2 | 100.0 | 155.0 | 18 | 12 | 2.80 | - | - | - |
| Kynurenine | 209.1 | 94.0 | 118.0 | 18 | 12 | 2.81 | 20.9 | 63.4 | Kynurenine-13C10 |
| Serotonin | 177.1 | 160.0 | 115.0 | 26 | 16 | 2.88 | 2.5 | 7.7 | Tryptophan-d5 |
| Hydroxyanthranilic Acid-d3 | 157.1 | 139.0 | 83.0 | 24 | 12 | 3.56 | - | - | - |
| 3-Hydroxyanthranilic Acid | 154.0 | 136.0 | 80.0 | 24 | 12 | 3.60 | 1.4 | 4.8 | Hydroxyanthranilic Acid-d3 |
| Indoxylsulfate (NEG) | 212.1 | 80.0 | 132.0 | 36 | 15 | 3.64 | 39.0 | 118.2 | Tryptophan-d5 |
| Tryptophan-d5 (NEG) | 208.2 | 121.0 | 164.0 | 30 | 18 | 3.94 | - | - | - |
| Tryptophan-d5 | 210.2 | 151.0 | 123.0 | 32 | 16 | 3.95 | - | - | - |
| Tryptophan | 205.1 | 146.0 | 118.0 | 32 | 16 | 3.99 | 351.1 | 1064.0 | Tryptophan-d5 |
| Xanthurenic Acid | 206.0 | 132.0 | 188.0 | 30 | 32 | 4.05 | 13.5 | 40.8 | Kynurenic Acid-d5 |
| Kynurenic Acid-d5 | 195.1 | 149.0 | 94.0 | 6 | 22 | 4.35 | - | - | - |
| Kynurenic Acid | 190.0 | 144.0 | 89.0 | 6 | 22 | 4.38 | 2.5 | 7.6 | Kynurenic Acid-d5 |
| 5-Hydroxyindoleacetic Acid | 192.1 | 146.0 | 91.0 | 24 | 16 | 4.60 | 2.4 | 7.2 | 5-Hydroxyindoleacetic Acid-13C6 |
| 5-Hydroxyindoleacetic Acid-13C6 | 198.1 | 152.0 | 96.0 | 30 | 20 | 4.60 | - | - | - |
| Tryptamine-d4 | 165.2 | 148.0 | 118.0 | 12 | 10 | 4.62 | - | - | - |
| Tryptamine | 161.1 | 144.0 | 127.0 | 4 | 24 | 4.65 | 709.1 | 2148.8 | Tryptamine-d4 |
| Indoleacetaldehyde | 160.1 | 132.0 | 77.0 | 34 | 14 | 5.02 | 16.8 | 51.0 | Indoleacetic Acid-d7 |
| Acetylserotonin | 219.1 | 160.0 | 115.0 | 38 | 14 | 5.02 | 0.3 | 0.8 | 5-Hydroxyindoleacetic Acid-13C6 |
| Indoleacetamide | 175.1 | 130.0 | 77.0 | 28 | 14 | 5.55 | 0.6 | 1.9 | Indoleacetic Acid-d7 |
| Indolelactic Acid | 206.1 | 118.0 | 130.0 | 30 | 20 | 6.32 | 161.4 | 489.1 | Indoleacetic Acid-d7 |
| Acetyltryptophan | 247.1 | 201.0 | 205.0 | 40 | 12 | 6.49 | 1.9 | 5.7 | 5-Hydroxyindoleacetic Acid-13C6 |
| Indole | 118.1 | 91.0 | 65.0 | 10 | 24 | 6.60 | 2.2 | 6.8 | Indoleacetic Acid-d7 |
| Indolealdehyde | 146.1 | 118.0 | 91.0 | 26 | 16 | 6.60 | 1.0 | 3.1 | Indoleacetic Acid-d7 |
| Indoleacetic Acid-d7 | 183.1 | 136.0 | 108.0 | 30 | 12 | 6.80 | - | - | - |
| Indoleacetic Acid | 176.1 | 103.0 | 129.0 | 28 | 30 | 6.85 | 8.1 | 24.5 | Indoleacetic Acid-d7 |
| Tryptophol | 162.1 | 144.0 | 127.0 | 12 | 24 | 6.89 | 0.7 | 2.0 | Indoleacetic Acid-d7 |
| Melatonin-d3 | 236.2 | 174.0 | 130.0 | 36 | 14 | 6.91 | - | - | - |
| Melatonin | 233.1 | 174.0 | 130.0 | 42 | 14 | 6.92 | 0.4 | 1.2 | Melatonin-d3 |
| Indolepropionic Acid | 190.1 | 130.0 | 172.0 | 8 | 12 | 7.44 | 1.2 | 3.7 | Indoleacetic Acid-d7 |
| Indoleacrylic Acid | 188.1 | 170.0 | 115.0 | 26 | 16 | 7.46 | 9.6 | 29.2 | Indoleacetic Acid-d7 |
| Skatole | 132.1 | 117.0 | 90.0 | 30 | 20 | 8.13 | 13.1 | 39.8 | Indoleacetic Acid-d7 |
| Indolepyruvic Acid | 204.1 | 130.0 | 77.0 | 12 | 10 | 8.23 | 0.7 | 2.0 | Indoleacetic Acid-d7 |
